# Supplementary material for: The Interaction between Hydromulching and Arbuscular Mycorrhiza Improves Escarole Growth and Productivity by Regulating Nutrient Uptake and Hormonal Balance
Source: Plants (Basel). 2022 Oct 21;11(20):2795. doi: 10.3390/plants11202795 (PMC9612124; doi:10.3390/plants11202795)
Supplement: Supplementary file 1 [file plants-11-02795-s001.zip › plants-1964105-supplementary.pdf]

SUPPLEMENTARY FIGURE 1

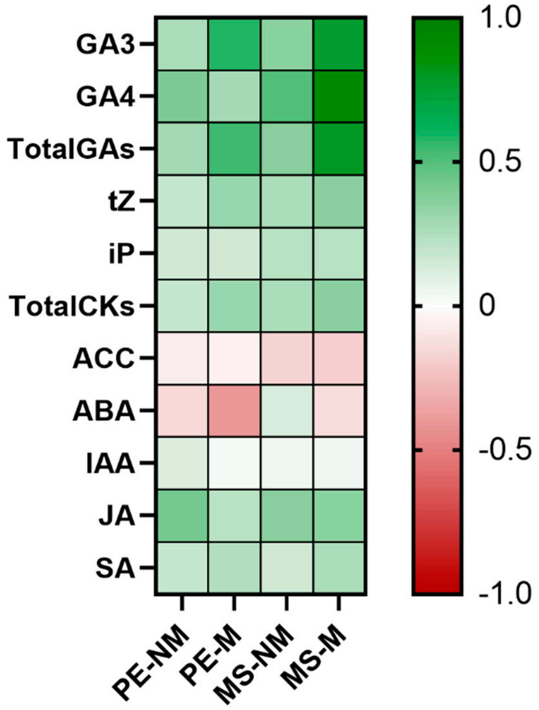

Figure S1. Cluster heatplot of the hormonal profile studied in escarole plants of the commercial variety “Bekele” non-mulched or subjected to different mulching treatments and cultivated under non-mycorrhizal (NM) and mycorrhizal (M) conditions. Abbreviations used: gibberellin A3 (GA3), gibberellin A4 (GA4), total gibberellins (Total GAs), *trans*-zeatin (tZ), isopentenyl adenine (iP), total cytokinins (Total CKs), 1-aminocyclopropane-1-carboxylic acid (ACC), abscisic acid (ABA), indole acetic acid (IAA), salicylic acid (SA), jasmonic acid (JA), polyethylene mulch (PE) and mushroom substrate-based hydromulch (MS).
